# MTX pathway gene variants, erythrocyte methotrexate polyglutamates, and treatment outcomes in rheumatoid arthritis

**DOI:** 10.17305/bb.2026.13544

**Published:** 2026-01-19

**Authors:** Peihong Wang, Cuilv Liang, Limei Lin, Jieli Lan, Yin Zhang, Weiping Xie

**Affiliations:** 1 Department of Pharmacy, The Second Affiliated Hospital of Fujian Medical University, Quanzhou, China; 2 Division of Science and Technology, The Second Affiliated Hospital, Fujian Medical University, Quanzhou, China; 3 The Affiliated Quanzhou Center for Disease Control and Prevention of Fujian Medical University, Quanzhou, China

**Keywords:** Rheumatoid arthritis, methotrexate, genetic polymorphisms, methotrexate polyglutamate

## Abstract

Rheumatoid arthritis (RA) exhibits significant inter-patient variability in response to and toxicity from methotrexate (MTX). The clinical utility of erythrocyte methotrexate polyglutamates (MTXPGs) and MTX-pathway pharmacogenetics remains uncertain. This study investigates the relationships between MTX-pathway gene polymorphisms, erythrocyte MTXPG levels, and MTX treatment outcomes in RA. In a single-center, cross-sectional cohort study conducted in southern Fujian from 2017 to 2020, we analyzed 140 Han Chinese RA patients who had been receiving stable low-dose oral MTX (7.5–15 mg/week) for at least three months. Genotyping was performed using MassARRAY, and MTXPG levels 1–6 were quantified in red blood cells via LC-MS/MS. Data on treatment efficacy (measured by ACR20 and clinical scales) and MTX-related adverse drug reactions (ADRs) were collected, with associations analyzed through univariate and multivariable models. MTXPG levels 1–3 were detectable in all patients, while longer-chain MTXPGs were infrequent. The *SLCO1B1* 521T>C polymorphism was independently associated with lower levels of MTXPG1 (B=−1.119), MTXPG2 (B=−0.924), and total MTXPG (B=−0.849), all with *P*-values ≤0.045. However, MTXPG levels did not correlate with MTX efficacy or ADRs. The *GGH* 401C>T polymorphism was associated with a reduced ACR20 response (OR=0.421, *P* ═ 0.021) and higher visual analog scale (VAS) and patient global assessment (PGA) scores. Additionally, the variants *SLCO1B1* 521T>C and *ABCB1* 3435C>T were linked to higher scores in the Patient Health Global Assessment (PHGA) and Health Assessment Questionnaire (HAQ). In this low-dose MTX cohort, erythrocyte MTXPGs did not predict clinical outcomes. However, variants in *SLCO1B1*, *GGH*, and *ABCB1* emerged as exploratory candidate markers for MTX response, warranting validation in larger prospective cohorts.

## Introduction

Rheumatoid arthritis (RA) is a chronic systemic inflammatory disease that can damage joints and affect multiple extra-articular organs [1]. The global age-standardized prevalence and incidence rates of RA have shown significant variation over time. If left untreated, RA symptoms can severely impair a patient’s ability to work and perform daily activities [2]. Due to its efficacy, safety profile, affordability, and flexible administration, methotrexate (MTX) is recommended as a first-line treatment for RA in several clinical guidelines [3–5]. However, MTX exhibits considerable inter-patient variability, with 30%–50% of patients failing to achieve remission and up to 30% experiencing adverse drug reactions (ADRs) that necessitate discontinuation due to toxicity [6, 7]. Numerous studies have suggested that individual differences in drug response may be linked to intracellular and plasma drug concentrations [8, 9]. Unfortunately, accurately monitoring serum MTX concentrations in RA patients is challenging due to its rapid decline in plasma [10]. Direct monitoring of MTX concentrations has proven unreliable for predicting clinical outcomes, and the search for more robust biomarkers of MTX treatment response remains ongoing.

In RA treatment, a small weekly dose of MTX (7.5–20.0 mg) is commonly used. When administered subcutaneously or orally, MTX is transported into cells and undergoes polyglutamation to form methotrexate polyglutamates (MTXPGs). γ-Glutamyl hydrolase deconjugates MTXPGs in a competing reaction, yielding a range of chain lengths (MTXPG1-6) [11]. MTXPGs are the active intracellular metabolites of MTX, with an average half-life of approximately 1–4 weeks, during which they continuously exert anti-rheumatic effects [12]. MTXPGs accumulate in red blood cells (RBCs) and are easily detectable. Several methods can be employed to determine MTXPG levels in RBCs, among which the recently described liquid chromatography-tandem mass spectrometry (LC-MS/MS) method can directly measure individual concentrations of MTXPG1-6 [13]. Studies have indicated that MTXPGs correlate with drug efficacy and may serve as potential biomarkers for RA treatment response [14, 15]. However, ongoing discussions exist regarding the predictive value of MTXPG levels for efficacy and safety in RA patients, potentially due to sample size limitations, ancestry heterogeneity, and other confounding factors [14, 16].

**Figure 1.**
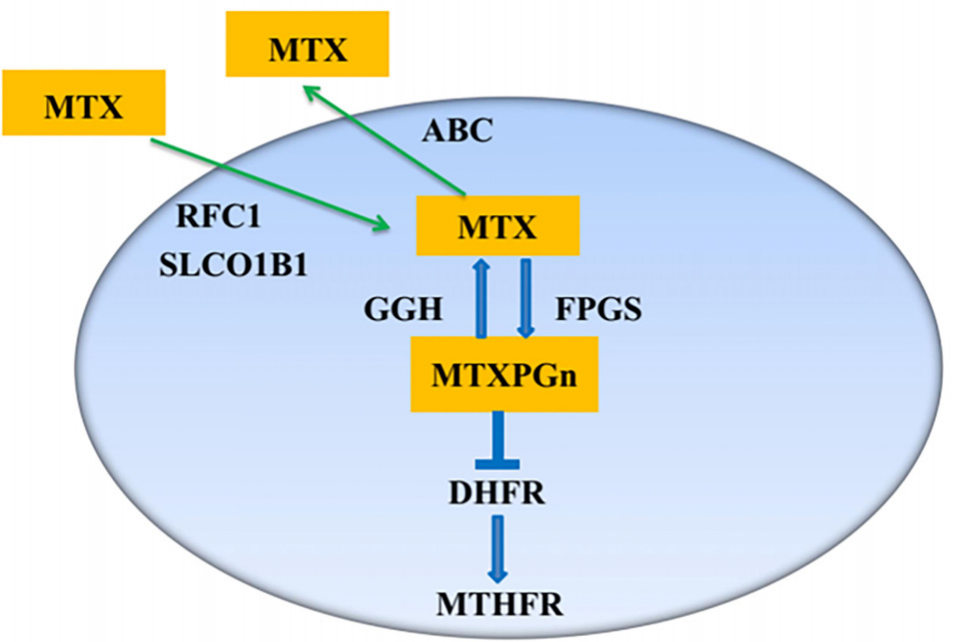
**Schematic overview of MTX transport, intracellular MTXPGn formation, and key steps in the MTX folate pathway.** MTX enters cells via RFC1 and SLCO1B1 and is converted by FPGS into MTXPGn. MTXPGn can be deglutamated by GGH back to MTX, which may be exported by ABC transporters. MTXPGn inhibit DHFR, thereby perturbing downstream folate/one-carbon metabolism involving MTHFR. Polyglutamate chain length is indicated by n. Abbreviations: MTX: Methotrexate; MTXPGn: Methotrexate polyglutamates; RFC1: Reduced folate carrier 1; SLCO1B1: Solute carrier organic anion transporter 1B1; FPGS: Folylpolyglutamate synthase; GGH: Gamma-glutamyl hydrolase; ABC: ATP-binding cassette; DHFR: Dihydrofolate reductase; MTHFR: Methylenetetrahydrofolate reductase.

As illustrated in Figure 1, several key metabolic enzymes are involved in the transport and metabolism of MTX. MTX is transported into cells via Reduced Folate Carrier 1 (RFC1) and Solute Carrier Organic Anion Transporter 1B1 (SLCO1B1). Once inside, MTX is catalyzed by Folylpolyglutamate Synthase (FPGS) to produce MTXPGs, which are subsequently hydrolyzed by Gamma-Glutamyl Hydrolase (GGH) back to the parent compound. When MTXPGs are converted back to MTX by GGH, the drug is quickly transported out of the cell by the ATP-Binding Cassette (ABC) family pump. MTXPGs inhibit Dihydrofolate Reductase (DHFR), thereby preventing the reduction of dihydrofolate to tetrahydrofolate, a reaction catalyzed by Methylenetetrahydrofolate Reductase (MTHFR) during one-carbon unit transfer. Several studies have reported that genetic polymorphisms in MTX transport and metabolism genes influence treatment responses in RA patients, although some findings have been paradoxical [17–24]. Limited studies are available regarding the impact of genetic polymorphisms in methotrexate pathway genes (GPMTX) on intracellular MTXPG levels, and the findings remain inconsistent [25–28]. For example, Ando et al. reported that RFC1 80G>A was significantly associated with the detectability of MTXPG5, whereas an Indian study found no association between this variant and MTXPG levels. Consequently, the relationship between GPMTX and MTXPG levels remains unclear [27, 28]. Therefore, we selected nine single nucleotide polymorphisms (SNPs) from eight key genes involved in the transport and metabolism of MTX, which are well-studied yet controversial, and measured MTXPG levels in RBCs using the more specific and sensitive LC-MS/MS method to clarify their respective influences on MTX response and to delineate the genotype-metabolite relationship in RA patients.

## Materials and methods

### Patients

This study enrolled 140 RA patients undergoing stable oral MTX pulse therapy at a tertiary hospital from October 2017 to July 2020. Patients were included based on the following criteria: they were from southern Fujian and of Han nationality; diagnosed with RA according to the 2010 American College of Rheumatology/European League Against Rheumatism (ACR/EULAR) guidelines [29]; had received stable oral MTX therapy (7.5–15.0 mg once weekly for ≥3 months, with stable concentrations of MTX in RBCs); and maintained stable concomitant medications for ≥4 weeks before enrollment. Hepatic function had to be Child-Pugh class A or B with transaminases ≤ 2 × the upper limit of normal, and renal function required an estimated glomerular filtration rate (eGFR) ≥ 60 mL/min/1.73 m^2^. Exclusion criteria included patients receiving concomitant medications known to alter MTX pharmacokinetics, those with significant cardiac, hepatic, pulmonary, or renal disease, or individuals unable or unwilling to provide informed consent.

In our study, the sample size was calculated based on the principle of “10 events per variable” [30, 31]. Treatment adherence was an inclusion criterion, and a 10% dropout rate was assumed in the sample size calculation. Forward calculation estimated the required sample size to be 67–156 cases. In back-calculation validation, 23–78 cases were needed for linear regression and generalized linear models, ensuring favorable statistical power for our study.

### Data collection

Demographic and medication information was collected using online electronic systems. A standard data-collection form recorded each patient’s name, sex, age, ethnicity, height, weight, time from last dose to sampling, treatment regimen, and past medication history. Efficacy was evaluated using the American College of Rheumatology 20 (ACR20) diagnostic criteria for RA, categorizing patients into effective and ineffective groups. Several widely used efficacy evaluation scales, including tender joint count 28 (TJC28), swollen joint count 28 (SJC28), visual analogue scale (VAS), patients’ global assessment (PGA), physicians’ global assessment (PHGA), and health assessment questionnaire (HAQ), were also utilized as complementary methods for evaluation. MTX-related ADRs were assessed based on the causality criteria of the National Adverse Drug Reaction Monitoring Centre. Patients whose ADRs were rated as “definite,” “very likely,” or “possible” were assigned to the ADR group, while those rated as “possibly unrelated,” “to be evaluated,” or “unable to evaluate” constituted the non-ADR group. In this cross-sectional study, the ACR20 response was evaluated as a composite outcome measure based on changes from baseline. The assessment period extended from the initiation of MTX therapy in patients to the date of MTXPG measurement. All participants were required to have maintained a stable dose of MTX for at least three months prior to the ACR20 assessment, ensuring that MTXPG levels had reached a steady state. Concurrent with the ACR20 assessment, baseline patient characteristics, concomitant medications, and occurrences of ADRs were collected. ADRs were captured through medical record reviews, in-person interviews, and questionnaires. Hepatotoxicity was defined as an elevation in aspartate aminotransferase or alanine aminotransferase levels exceeding the upper limit of the laboratory’s normal reference range. A patient was considered to have experienced hepatotoxicity during the period (from the initiation of MTX administration to the date of MTXPG measurement) if they met the hepatotoxicity criteria at least once within that timeframe.

### Genetic analysis

The genomic DNA of each subject was extracted from 4 mL of peripheral venous blood stored in an EDTA-coated tube at –80 ^∘^C. The MassARRAY method, which utilizes time-of-flight mass spectrometry for direct SNP typing, was employed to detect and analyze the distribution of nine loci: *GGH 401C>T*, *ABCC2 24 C>T*, *MTHFR 677C>T*, *ABCB1 3435C>T*, *ABCC4 (rs9516519)*, *MTHFR 1298A>C*, *RFC1 80G>A*, *FPGS G>A*, and *SLCO1B1 521T>C* in RA patients from southern Fujian. Following the manufacturer’s protocol, a specialized DNA extraction kit was used for the extraction from EDTA blood samples. DNA quality was assessed via electrophoresis of 5 µL aliquots on 1% agarose gels in 1× TAE buffer at 120–180 V for 15 min. A single, sharp band indicated high-molecular-weight, non-degraded DNA of sufficient concentration for subsequent Polymerase Chain Reaction (PCR) (Figure S1). The MALDI-TOF System (Sequenom, USA) was utilized for SNP genotype detection to obtain genotype data.

### Monitoring of MTXPG levels

For sample pre-treatment, milli-Q water was used to lyse red blood cells in EDTA-anticoagulated whole blood, followed by protein precipitation with perchloric acid [32]. The complete sample pretreatment procedure involved mixing 200 µL of thawed whole blood with 400 µL of ultrapure water for red blood cell lysis, followed by the addition of 400 µL of a 4% perchloric acid aqueous solution. The mixture was vortexed and centrifuged at 10,000 rpm for 5 min. The supernatant was loaded onto an Oasis MAX solid-phase extraction cartridge preconditioned with 1 mL of methanol and 1 mL of water. After sample loading, the cartridge was washed with 1 mL of 5% ammonia and then with 1 mL of 100% methanol. The analyte was eluted using 0.8 mL of a methanol-water (6:4, v/v) solution containing 2% formic acid. The eluate was dried under a gentle stream of nitrogen at 40 ^∘^C. The residue was reconstituted in 100 µL of 0.1% NH_4_OH, centrifuged at 10,000 rpm for 5 min, and the supernatant was transferred to an autosampler vial for LC-MS analysis.

As reported by Hawwa et al., haematocrit introduced <5% error into MTXPG concentration measurements, rendering the influence of haematocrit and RBC count negligible [33]. These authors also demonstrated that MTXPGn remained stable at –80 ^∘^C and 25^∘^C for two months, confirming adequate stability for our analytical procedure; thus, no in-house stability testing was performed [33]. Steady-state concentrations of MTXPG1-6 in RBCs were quantified by LC-MS/MS. The performance parameters of the MTXPG analytical method were validated according to the FDA (Bioanalytical Method Validation Guidance for Industry) and EMEA (Guideline on bioanalytical method validation) guidelines (Table S1). Analyses included (1) determination of detection limits and quantification limits; (2) measurement of recovery and precision (Table S2); and (3) correlation coefficients and linear ranges of the standard curves for MTXPG (Table S3). The liquid chromatography-mass spectrometry (LC-MS) system employed was the AB SCIEX 4000+ liquid chromatography-tandem mass spectrometer (AB SCIEX, Concord, Ontario, Canada) with an electrospray ionization source. MTXPG1-6 standards were procured from Schircks Laboratories (Jona, Switzerland). The lot numbers for the MTXPG1-6 standards were as follows: MTXPG1 (16.411 lot 014), MTXPG2 (16.412 lot 105), MTXPG3 (16.413 lot 17), MTXPG4 (16.414 lot 12), MTXPG5 (16.415 lot 15), and MTXPG6 (16.416 lot 31). The purity of these standards met the specified requirements. Ammonium bicarbonate, LC-MS-grade methanol, and formic acid were obtained from TEDIA (Fairfield, USA), while perchloric acid and acetic acid were sourced from Sinopharm Chemistry Reagent Co. Ltd (Shanghai, China). Chromatography was performed on 10 µL aliquots following partial-loop injection, utilizing a Beckman C8 column (4.6×250 mm, 5 µm) (Beckman, CA, USA) maintained at 35^∘^C. The mobile phase consisted of (A) 10 mM ammonium bicarbonate buffer adjusted to pH 10 with 25% ammonia solution and (B) methanol at a flow rate of 0.5 mL/min. The elution program was as follows: 0–0.5 min isocratic hold 5% B, 0.5–6.0 min linear gradient 5%–60% B; 6.0–6.5 min isocratic 60% B; 6.5–7.5 min linear gradient 60%–100% B; 7.5–9.0 min isocratic 100% B; 9.0–10.0 min linear gradient 100%–5% B; and 10.0–15.0 min isocratic 5% B. The electrospray ionization source operated in positive-ion mode, and quantification was performed via multiple-reaction monitoring. The monitoring ion pairs for MTXPG1-6 were as follows: MTXPG1: 455.2/308.2; MTXPG2: 584.3/308.2; MTXPG3: 713.2/308.2; MTXPG4: 842.5/308.2; MTXPG5: 486.6/308.2; MTXPG6: 550.9/308.2. In calculating MTXPG levels, concentrations below the limit of detection (LOD) were treated as zero, while concentrations ≥ LOD and < limit of quantification (LOQ) were included at their measured values.

### Statistical analysis

Data were analyzed using SPSS 22.0 software (IBM Corporation, Armonk, NY, USA). Initially, Hardy-Weinberg equilibrium was tested to verify that the distribution of gene polymorphisms was consistent with a genetically balanced population. Subsequently, univariate analysis was conducted to analyze continuous and categorical variables. Numerical data were presented as mean ± standard deviation (SD) or median (interquartile range) and assessed for normality using the Kolmogorov-Smirnov test. For normally distributed data, *t*-tests or analysis of variance were utilized for group comparisons. For non-normally distributed data, Spearman’s rank correlation analysis was employed for correlation analysis, and the Mann-Whitney *U* test or Kruskal-Wallis test was applied for group comparisons. Chi-square test or Fisher’s exact test were used for categorical data comparisons. Finally, variables with *P* < 0.20 in the univariate analysis were incorporated into multiple linear regression, binary logistic regression, or generalized linear models to investigate the relationship between GPMTX and MTXPG levels and their impact on RA treatment response. A two-sided *P*-value < 0.05 was established as the significance threshold for this study.

### Ethical statement

This study was registered with the Chinese Clinical Trial Registry and approved by the local Internal Ethics Review Board (Ethics No. 41||2018-05-20). The study was conducted in accordance with the guidelines of the Declaration of Helsinki, and all patients provided informed consent prior to participation.

## Results

### Basic patient information

A total of 140 participants, comprising 36 males and 104 females, were recruited from RA patients in the southern Fujian region, with a median age of 50 years, median height of 160 centimeters, and median weight of 55 kilograms. There were no significant differences in demographic characteristics between the effective and ineffective groups (Table 1).

**Table 1:** Baseline demographic characteristics of patients with rheumatoid arthritis

**Characteristics**	**All patients (*n* ═ 140)**	**Effective (*n* ═ 60)**	**Ineffective (*n* ═ 80)**	***P* value**
Age, years, mean ± SD	50.0±12.0	48.8±12.3	50.2±11.3	0.504
Height, cm, median (IQR)	160 (155.0--165.0)	160 (155.0--166.5)	158 (155.0--163.8)	0.246
Weight, kg, median (IQR)	55 (49.3--63.4)	55.25 (50.0--63.8)	55 (48.6--63.4)	0.956
Gender, male (%)	36 (25.7)	15 (25.0)	21 (26.3)	0.867
Time^*^, hour, median (IQR)	4.5 (3.0--6.8)	4.75 (3.0--7.0)	4.5 (2.0--6.0)	0.610
Dose^+^, mg/week, median (IQR)	10 (10--10)	10 (10--10)	10 (10--10)	0.506
Course^#^, years, median (IQR)	1 (0.3--4.9)	1 (0.3--3.1)	1 (0.3--5.0)	0.445
Duration^§^, month, median (IQR)	5.5 (3.0--13. 8)	6 (3.0--14.8)	4 (3.0--12.0)	0.116

^*^Time refers to the interval between the last dose and sampling after achieving steady-state concentrations, which occurs under stable dosing following a minimum of three months.^+^Dose, the dosage of the drug; ^#^Course, the course of the disease; ^§^Duration, the duration of the drug. Abbreviations: SD: Standard deviation; IQR: Inter quartile range; RA: Rheumatoid arthritis.

### Gene distribution

In this study, the success rate for sample genotyping was 100%, with no duplicate genotyped samples identified. Except for the *ABCC4* variant (rs9516519), which was not detected, and *ABCC2 24C>T*, which deviated from Hardy-Weinberg equilibrium (X^2^ ═ 8.451, *P* < 0.05), all remaining polymorphisms were found to be in equilibrium. Therefore, ABCC4_ (rs9516519) and *_ABCC2 24C>T* were excluded from further analysis. A comparison of our results with the PharmaGKB and NCBI SNP databases revealed that the variant frequencies of *GGH 401T*, *MTHFR 677T*, *ABCB1 3435T*, *MTHFR 1298C*, *RFC1 80A*, *FPGS A*, and *SLCO1B1 521C* in the southern Fujian population were similar to those observed in the East Asian population.

**Figure 2.**
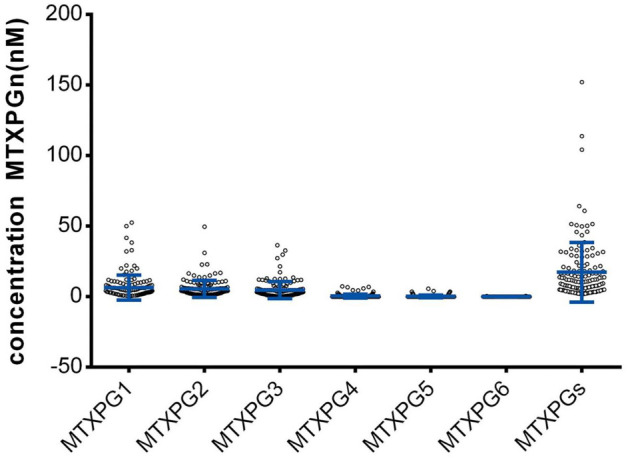
**Distribution of erythrocyte MTXPG concentrations.** MTXPG1–3 were detected in all patients (ranges: MTXPG1 0.430–52.700 nM; MTXPG2 0.980–49.700 nM; MTXPG3 0.482–36.500 nM). MTXPG4 was detected in 19 patients (12 results ≥LOD and <LOQ; 7 results 3.56–7.370 nM). MTXPG5 was detected in 10 patients (9 results ≥LOD and <LOQ; 1 result 5.740 nM). MTXPG6 was detected in 3 patients (all results ≥LOD and <LOQ). Total MTXPGs ranged from 1.762–152.273 nM, and 135 patients had total MTXPGs <60 nM. Dots represent individual patients; blue bars indicate mean ± SD. Abbreviations: MTXPG: Methotrexate polyglutamate; MTXPGs: Total methotrexate polyglutamates; LOD: Limit of detection; LOQ: Limit of quantification; SD: Standard deviation.

### MTXPG levels

MTXPG1, MTXPG2, and MTXPG3 were detected in all patients (concentration range: MTXPG1 0.430–52.700 nmol/L; MTXPG2 0.980–49.700 nmol/L; MTXPG3 0.482–36.500 nmol/L). MTXPG4 was detected in 19 patients (12 results were above the LOD but below the LOQ, while the remaining 7 results were within the concentration range of 3.56–7.370 nmol/L). MTXPG5 was detected in 10 patients (9 results were above the LOD but below the LOQ, while only 1 result exceeded the LOQ, with a concentration of 5.740 nmol/L). MTXPG6 was detected in 3 patients (all results were above the LOD but below the LOQ). The total concentration range of MTXPGs was 1.762–152.273 nmol/L, with 135 patients exhibiting MTXPG levels lower than 60 nmol/L (Figure 2).

**Figure 3.**
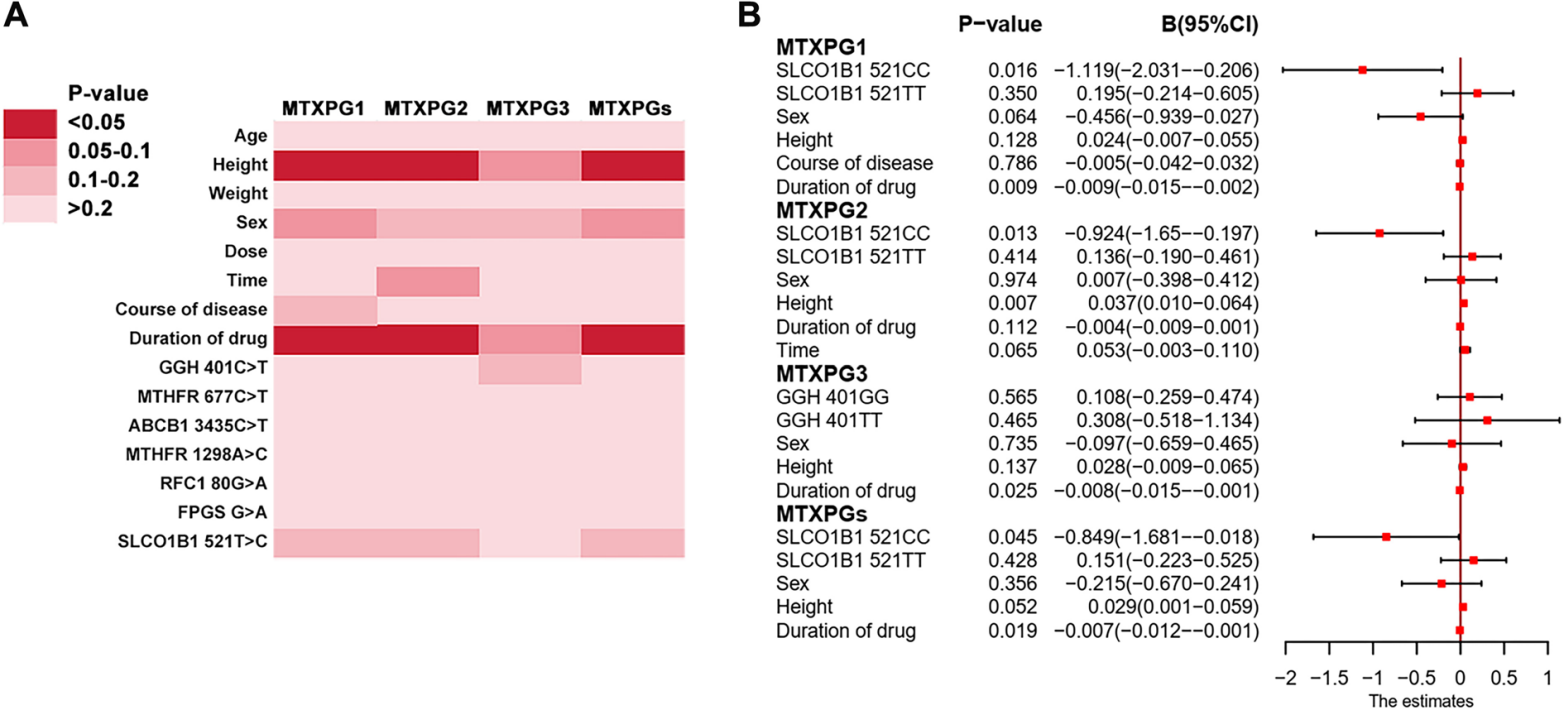
**Associations of MTX-pathway SNPs and clinical covariates with erythrocyte MTXPG concentrations.** (A) Heatmap summarizing univariate analyses between demographic/treatment variables and seven MTX-pathway SNPs versus MTXPG1, MTXPG2, MTXPG3 and total MTXPGs; colour intensity denotes the P-value category (scale shown). (B) Forest plot of covariate-adjusted effects from outcome-specific generalized linear models; points indicate regression coefficients (B) and whiskers the 95% CI. Consistent with the multivariable models, *SLCO1B1* 521T>C was significantly associated with MTXPG1, MTXPG2 and total MTXPGs. Genotypes were modelled as categorical variables, and B estimates represent differences relative to the reference genotype. Abbreviations: MTXPG: Methotrexate polyglutamate; MTXPGs: Total methotrexate polyglutamates; SNP: Single nucleotide polymorphism; B: Regression coefficient; CI: Confidence interval; GGH: Gamma-glutamyl hydrolase; MTHFR: Methylenetetrahydrofolate reductase; ABCB1: ATP-binding cassette subfamily B member 1; RFC1: Reduced folate carrier 1; FPGS: Folylpolyglutamate synthase; SLCO1B1: Solute carrier organic anion transporter 1B1.

### Correlation of GPMTX with MTXPG levels

This study analyzed the effects of seven SNPs in MTX-pathway genes on intracellular MTXPG levels. Univariate and multivariate analyses were conducted with genotype and clinical parameters as independent variables, and MTXPG1, MTXPG2, MTXPG3, and total MTXPG levels as dependent variables. As illustrated in Figure 3, the genotype of SLCO1B1 521T>C was significantly correlated with MTXPG1 (regression coefficient (B) ═ –1.119, 95% confidence interval: –2.301 to –0.206, *P* ═ 0.016), MTXPG2 (B ═ –0.924, 95% CI: –1.65 to –0.197, *P* ═ 0.013), and total MTXPG (B ═ –0.849, 95% CI: –1.681 to –0.018, *P* ═ 0.045) in generalized linear models. The diagnostic plot of standardized residuals against predicted values indicated that the standardized residuals were randomly distributed around the zero line, with no discernible trend or heteroscedastic pattern, suggesting that the assumption of homogeneity of variance was largely satisfied. The influence diagnostic plot showed that all Cook’s distances remained well below the threshold of 1.0 for strong influential points, indicating stability and reliability in the model diagnostic results (Figure S2-S7).

### Correlation of MTXPG levels with efficacy and ADR of MTX in RA patients

**Figure 4.**
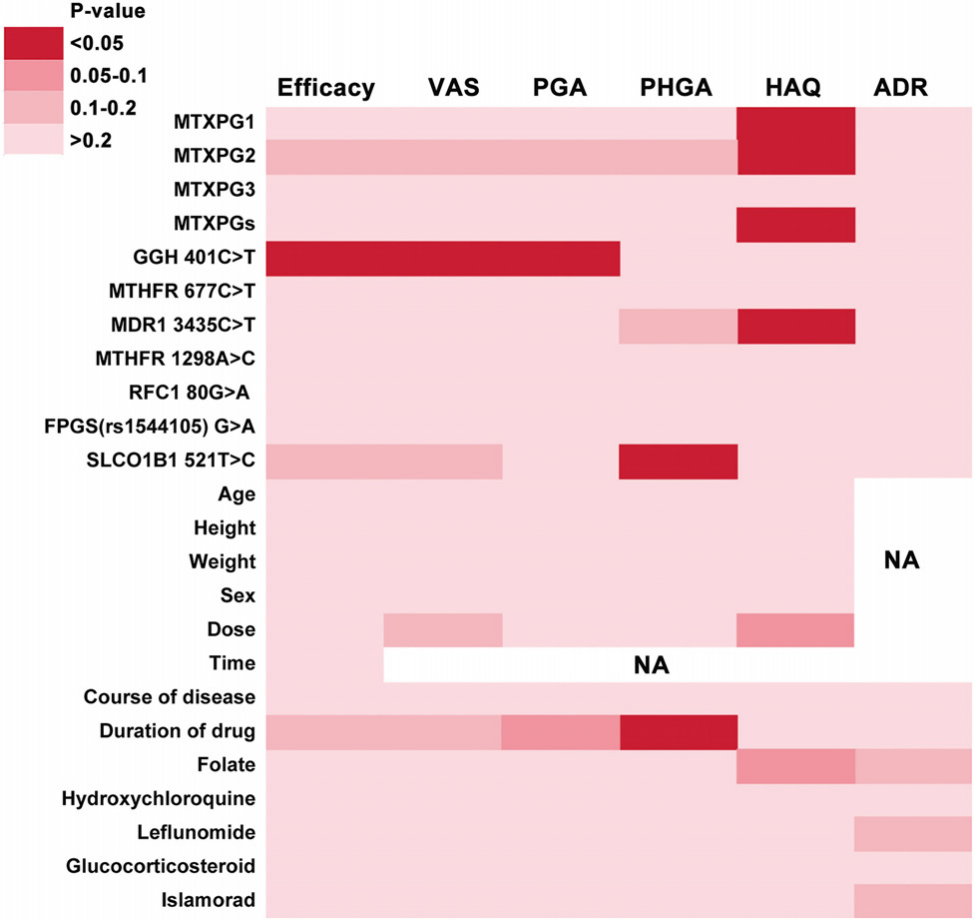
**Heatmap summary of univariate associations between erythrocyte MTXPG measures, MTX-pathway SNPs, and clinical covariates with MTX treatment outcomes in RA.** Columns show efficacy (ACR20 responder status), VAS, PGA, PHGA, HAQ, and MTX-related ADRs; rows include MTXPG1–3 and total MTXPGs, seven MTX-pathway SNPs, demographic/treatment variables, and concomitant medications. Cell shading denotes *P* value categories (key shown); NA indicates outcomes not assessed for the corresponding predictor. In univariate testing, MTXPG1, MTXPG2 and total MTXPGs were significantly associated with HAQ, *GGH* 401C>T with efficacy as well as VAS and PGA, *ABCB1* (MDR1) 3435C>T with HAQ, and *SLCO1B1* 521T>C with PHGA; no significant associations were observed for ADRs. Abbreviations: RA: Rheumatoid arthritis; MTX: Methotrexate; MTXPG: Methotrexate polyglutamate; MTXPGs: Total methotrexate polyglutamates; ACR20: American College of Rheumatology 20% response; VAS: Visual analogue scale; PGA: Patient’s global assessment; PHGA: Physician’s global assessment; HAQ: Health Assessment Questionnaire; ADR: Adverse drug reaction; SNP: Single nucleotide polymorphism; NA: Not applicable; GGH: Gamma-glutamyl hydrolase; MTHFR: Methylenetetrahydrofolate reductase; ABCB1/MDR1: ATP-binding cassette subfamily B member 1 (multidrug resistance 1); RFC1: Reduced folate carrier 1; FPGS: Folylpolyglutamate synthase; SLCO1B1: Solute carrier organic anion transporter 1B1.

In this study, 35 patients (25%) experienced ADRs, with the most common being nausea/vomiting (*n* ═ 12), abdominal distension (*n* ═ 5), dizziness (*n* ═ 4), and hepatotoxicity (*n* ═ 4). The Mann-Whitney *U* test was applied for group comparisons, and Spearman’s rank correlation was utilized to examine the association of MTXPG1-3 and total MTXPG levels with MTX efficacy, as evaluated by efficacy scales. Significant correlations were found between RBC levels of MTXPG1 (*P* ═ 0.015), MTXPG2 (*P* ═ 0.025), and total MTXPG (*P* ═ 0.048) with the HAQ score (Figure 4).

### Correlation of GPMTX with efficacy and ADRs of MTX in RA patients

Univariate and multivariate analyses were performed to evaluate the effects of seven SNPs in MTX pathway genes, along with demographic characteristics and concomitant medications, on the efficacy and ADRs of MTX. In the univariate analyses (Figure 4), the genotypes of *GGH 401C>T* influenced efficacy (Fisher’s exact test: *P* ═ 0.045), Visual Analog Scale (VAS) scores (*P* ═ 0.010), and PGA scores (*P* ═ 0.043)*. The ABCB1 3435C>T* genotype was associated with HAQ scores (*P* ═ 0.045), *while SLCO1B1 521T>C* impacted PHGA scores (*P* ═ 0.026). No significant associations were found for the remaining genotypes concerning efficacy or ADR groups.

### Multivariate analyses of GPMTX and MTXPG levels with efficacy of MTX in RA patients

**Figure 5.**
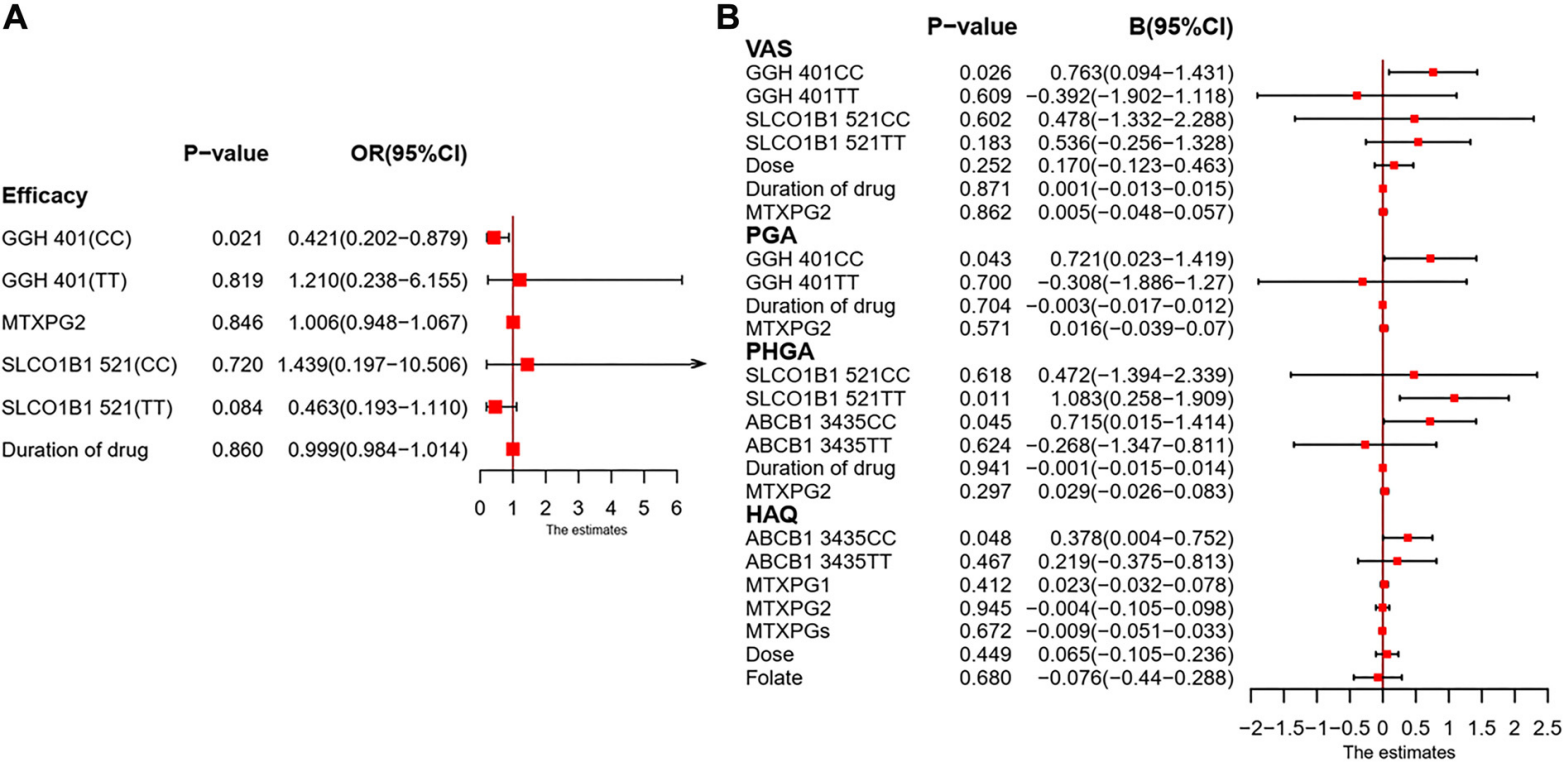
**Multivariable associations of MTX-pathway genotypes and erythrocyte MTXPG concentrations with MTX efficacy in RA.** (A) Logistic regression model for ACR20 response (events: 60/140), including *GGH* 401C>T, *SLCO1B1* 521T>C, MTXPG2 and duration of drug; results are shown as OR with 95% CI. (B) Multiple linear regression models for efficacy scales, shown as B with 95% CI: VAS (*GGH* 401C>T, *SLCO1B1* 521T>C, dose, duration of drug, MTXPG2), PGA (*GGH* 401C>T, duration of drug, MTXPG2), PHGA (*SLCO1B1* 521T>C, *ABCB1* 3435C>T, duration of drug, MTXPG2), and HAQ (*ABCB1* 3435C>T, MTXPG1, MTXPG2, MTXPGs, dose, folate). Genotypes are displayed as homozygous groups (CC or TT) relative to the heterozygous reference (CT). Squares indicate point estimates and horizontal lines the 95% CI; vertical lines denote the null effect (OR=1 in A; B=0 in B). Abbreviations: RA: Rheumatoid arthritis; MTX: Methotrexate; MTXPG: Methotrexate polyglutamate; MTXPGs: Total methotrexate polyglutamates; ACR20: American College of Rheumatology 20% response; OR: Odds ratio; B: Regression coefficient; CI: Confidence interval; VAS: Visual analogue scale; PGA: Patient’s global assessment; PHGA: Physician’s global assessment; HAQ: Health Assessment Questionnaire; GGH: Gamma-glutamyl hydrolase; SLCO1B1: Solute carrier organic anion transporter 1B1; ABCB1: ATP-binding cassette subfamily B member 1.

In multivariate analyses, a logistic regression model (Figure 5A, events: 60/140) indicated that *GGH 401C>T* contributed to differences in efficacy (odds ratio (OR) ═ 0.421, 95% CI: 0.202–0.879, *P* ═ 0.021). MTXPG2 levels did not significantly correlate with MTX efficacy (OR = 1.006, 95%CI: 0.948–1.067, *P* ═ 0.846). A multiple linear regression model (Figure 5B) showed that *GGH 401C>T* impacted VAS scores (*B* ═ 0.763, 95% CI: 0.094–1.431, *P* ═ 0.026) and PGA scores (*B* ═ 0.721, 95% CI: 0.023–1.419, *P* ═ 0.043)*. The SLCO1B1 521T>C* genotype affected PHGA scores (*B* ═ 1.083, 95% CI: 0.258–1.909, *P* ═ 0.011), *while ABCB1 3435C>T* influenced both PHGA scores (*B* ═ 0.715, 95% CI: 0.015–1.414, *P* ═ 0.045) and HAQ scores (*B* ═ 0.378, 95% CI: 0.004–0.752, *P* ═ 0.048). MTXPG2 levels were not correlated with VAS scores (*B* ═ 0.005, 95% CI: --0.048--0.057, *P* ═ 0.862), PGA scores (*B* ═ 0.016, 95% CI: --0.039--0.070, *P* ═ 0.571), or PHGA scores (*B* ═ 0.029, 95% CI: --0.026--0.083, *P* ═ 0.297). In the multiple linear regression model, significant associations of RBC levels of MTXPG1 (*B* ═ 0.023, 95% CI: –0.032-0.078, *P* ═ 0.412), MTXPG2 (B ═ –0.004, 95% CI: –0.105-0.098, *P* ═ 0.945), and total MTXPGs (B ═ –0.009, 95% CI: –0.051-0.033, *P* ═ 0.672) with the HAQ score were not observed. Since neither MTXPG levels nor the seven SNPs exhibited statistically significant associations with MTX-related ADRs in univariate analyses (*P* > 0.2), a multivariate model for ADRs was not constructed.

## Discussion

Previous research has debated whether GPMTX and MTXPG levels serve as stable biomarkers for predicting the efficacy and safety of MTX in RA patients. Additionally, the impact of GPMTX on MTXPG levels remains unclear. This prospective study aimed to thoroughly investigate these contentious relationships within a low-dose, single-center Han Chinese RA cohort. The following findings were observed: (1) The *SLCO1B1 521T>C* genotype may correlate with MTXPG levels in RBCs. (2) Under these constrained dose-exposure conditions, MTXPG levels may not predict the efficacy and safety of MTX in RA patients. (3) *GGH 401C>T, SLCO1B1 521T>*C, and *ABCB1 3435C>T* genotypes may serve as exploratory candidate markers for forecasting MTX efficacy.

Currently, there is limited exploration of the relationship between GPMTX and MTXPG levels in RA patients, resulting in ambiguous correlations. Apart from the SLCO1B1 521T>C genotype, no statistically significant associations were found between other SNPs and MTXPG levels in this study. A Japanese study indicated that *SLCO1B1 521T>C, RFC1 80G>A, ABCB1 3435C>*T, and *MTHFR 1298A>C* genotypes were not related to MTXPG levels in 55 Japanese RA patients receiving MTX monotherapy [16]. An Indian study also found no correlation between the genotypes of *ABCB1 3435C>T, FPGS G>A, GGH 401C>*T, and RFC1 80G>A with intracellular MTXPG levels in 117 RA patients [27]. Investigations involving East Asian populations generally suggested that most SNPs related to MTX transport and metabolism show no significant association with MTXPG levels. According to the PharmaGKB and NCBI SNP databases, the mutation frequencies of the genotypes studied here are similar to those in East Asian populations, which may serve as a reference. However, using a generalized linear model, this study found that *SLCO1B1 521T>C* significantly influenced MTXPG1, MTXPG2, and total MTXPG levels, contrasting with previous negative reports. This discrepancy may be attributed to two factors: the study population consisted exclusively of Han Chinese individuals, characterized by high ethnic homogeneity, potentially amplifying genetic effects specific to this ethnicity, and the use of LC-MS/MS for direct quantification of individual MTXPG1-6 subtypes within erythrocytes, providing higher sensitivity and specificity. It is essential to note that this study employed a cross-sectional, small-sample design. Although sample processing and quality control adhered to strict guidelines, further validation through large-sample, prospective cohort studies is necessary.

Furthermore, this study found no correlation between the concentrations of MTXPG1, MTXPG2, MTXPG3, and total MTXPG levels with therapeutic efficacy or ADRs in RA patients from the southern Fujian area. Regarding efficacy, several studies have reported results consistent with this study, while some have reported conflicting findings. The MIRACLE trial and a meta-analysis confirmed that elevated erythrocyte MTXPG concentrations correlated with decreased disease activity in RA [34, 35]. The MIRACLE trial was a 48-week randomized, open-label, parallel-group study involving 300 MTX-naïve patients who began treatment with oral MTX at 10 mg/week or 1 mg/day, escalating to the maximum tolerated dose by week 12 [34]. The meta-analysis included various immune-mediated inflammatory diseases and diverse study designs, with no restrictions on the MTX administration route [35]. The detection methods for MTXPG levels and regional populations in these studies also differed. This study employed a cross-sectional design, enrolling only Chinese patients who had maintained a stable oral MTX dose of 7.5–15.0 mg/week for at least three months. Given the low dose, single route of administration, and population homogeneity, no association between MTXPG levels and treatment response was detected in this investigation.

With respect to ADRs, the majority of studies align with our findings. There are two potential explanations for the lack of detected correlations in previous research. First, RA patients on low-dose MTX exhibit good long-term tolerability [36]. Second, concomitant medication is a significant influencing factor; RA patients frequently require combination therapy to manage disease activity. Strict restrictions on concomitant medications could substantially hinder enrollment feasibility and fail to accurately reflect real-world efficacy profiles. At enrollment, all concomitant medications were required to have been used stably for at least four weeks, indirectly controlling for fluctuations through medication stability. Additionally, we reanalyzed the impact of concomitant medications on toxicity and efficacy. Except for non-steroidal anti-inflammatory drugs (NSAIDs), no significant correlations were found between other concomitant medications and the efficacy or ADRs of MTX. Our analysis also revealed no correlation between GPMTX and MTXPG levels concerning MTX ADRs, indicating that NSAID use did not affect our study’s conclusions. However, existing literature suggests that folate supplementation may mitigate MTX-related ADRs, potentially influencing the relationship between MTXPG levels and ADRs [37]. Thus, a prospective study focusing on RA patients initially treated with MTX monotherapy is warranted to clarify this relationship.

Univariate and multiple analyses indicated correlations between *GGH 401C>T*, *SLCO1B1 521T>C*, and *ABCB1 3435C>T* and efficacy-related indicators. Notably, patients with the *GGH 401CC* genotype demonstrated an efficacy rate of only 46% compared to those with the *GGH 401CT* genotype. Previous studies have shown that SNPs in the GGH gene can influence GGH activity. The 401C>T polymorphism in the GGH promoter region *may enhance GGH* expression, thereby increasing GGH activity [38, 39]. GGH, encoded by the GGH gene, is an enzyme involved in the deglutamation of MTXPG, facilitating the elimination of MTXPG from cells. Our findings suggest that the CC genotype may increase GGH activity or expression compared to the CT genotype, potentially enhancing MTXPG elimination and reducing MTX efficacy. The *SLCO1B1* gene, expressed at the basolateral membrane of hepatocytes, facilitates the transport of MTX into cells. A double-blind, controlled study found that, compared to the wild-type group for *SLCO1B1 521T>C*, the area under the concentration-time curve of MTX increased 4.2-fold, and the peripheral clearance rate significantly decreased in the variant group [40]. Zhang et al. also reported a significant increase in plasma concentration of MTX compared to the wild-type group (*P* ═ 0.001) [41]. Thus, the wild-type group (*SLCO1B1 521 TT*) may exhibit decreased plasma concentrations of MTX, correlating with reduced MTX efficacy. MTX is transported out of cells by ABCB1, and meta-analysis indicates a significant association between *ABCB1 3435C>T* and MTX efficacy under a recessive model (CC vs. CT+TT; OR = 1.35; 95% CI: 1.01–1.82; *P* ═ 0.047) [18]. We also noted that the CC genotype was associated with increased MTX efficacy compared to patients with the CT genotype. These three genes are critical in the metabolism and elimination of MTX. Our study design incorporated demographic characteristics, allowing us to consider additional factors in our analyses, which may explain discrepancies between our findings and previous reports. Consequently, our study suggests that *GGH 401C>T*, *SLCO1B1 521T>C*, and *ABCB1 3435C>T* genotypes may serve as exploratory candidate markers for MTX efficacy in RA patients.

Several limitations of our study should be noted. First, while RBC sampling is practical, RBCs serve as surrogates and do not fully reflect intracellular MTXPG levels in synovial or lymphoid cells, which are more pharmacologically relevant. MTXPG4, MTXPG5, and MTXPG6 were detected in only a subset of patients, limiting our ability to explore correlations between their concentrations, gene polymorphisms, efficacy, and ADRs of MTX therapy in RA patients. Furthermore, only a single measurement of MTXPG levels was obtained, precluding repeated assessments during the patients’ follow-up period and limiting our ability to investigate variations in MTXPG levels over time. Third, due to cost and sample size constraints, confounding factors such as baseline disease activity, lifestyle habits, adherence (lacking objective evaluation), and potential biomarkers or genetic factors were not considered in our study, which could bias our interpretation of certain results. Lastly, our study was conducted in Han Chinese patients, raising questions regarding the generalizability of these findings to other ethnic groups.

## Conclusion

In summary, we utilized MassARRAY and LC-MS/MS methods to comprehensively investigate the relationship between GPMTX, MTXPG levels, and their effects on RA treatment. In this low-dose, single-center cohort of Han Chinese RA patients, we found no correlation between RBC MTXPG levels and GPMTX, nor with the efficacy and safety of MTX. This null finding may reflect a narrow dose-exposure window and should not be generalized to higher-dose populations. The genotypes *GGH 401C>T*, *SLCO1B1 521T>C*, and *ABCB1 3435C>T* may serve as exploratory candidate markers for MTX efficacy in this specific cohort, and the *SLCO1B1 521T>C* genotype may be associated with RBC MTXPG levels. These findings provide an experimental basis for the rational individualization of MTX therapy in RA patients. However, the relationship between MTXPG levels and MTX-related ADRs warrants further exploration, given the favorable tolerability of low-dose MTX and the influence of concomitant medications. Additionally, as this study represents a small-sample, single-center exploratory analysis, the current findings should be viewed as preliminary and warrant independent validation in larger-scale, prospective studies.

## Supplemental data

Supplemental data are available at the following link: https://www.bjbms.org/ojs/index.php/bjbms/article/view/13544/4105.

## Data Availability

The data sets obtained and/or analyzed during the current study are available from the corresponding author on reasonable request.

## References

[1] Radu AF, Bungau SG (2021). Management of rheumatoid arthritis: an overview. Cells.

[2] Uke P, Maharaj A, Adebajo A (2025). A review on the epidemiology of rheumatoid arthritis: an update and trends from current literature. Best Pract Res Clin Rheumatol.

[3] Smolen JS, Landewé RBM, Bergstra SA, Kerschbaumer A, Sepriano A, Aletaha D (2023). EULAR recommendations for the management of rheumatoid arthritis with synthetic and biological disease-modifying antirheumatic drugs: 2022 update. Ann Rheum Dis.

[4] Fraenkel L, Bathon JM, England BR, St Clair EW, Arayssi T, Carandang K (2021). 2021 American College of Rheumatology guideline for the treatment of rheumatoid arthritis. Arthritis Care Res (Hoboken).

[5] Fautrel B, Kedra J, Rempenault C, Juge PA, Drouet J, Avouac J (2024). 2024 update of the recommendations of the French Society of Rheumatology for the diagnosis and management of patients with rheumatoid arthritis. Joint Bone Spine.

[6] Smolen JS, Aletaha D (2015). Rheumatoid arthritis therapy reappraisal: strategies, opportunities and challenges. Nat Rev Rheumatol.

[7] Den Boer E, de Rotte MC, Pluijm SM, Heil SG, Hazes JM, de Jonge R (2014). Determinants of erythrocyte methotrexate polyglutamate levels in rheumatoid arthritis. J Rheumatol.

[8] Zijp TR, Izzah Z, Åberg C, Gan CT, Bakker SJL, Touw DJ (2021). Clinical value of emerging bioanalytical methods for drug measurements: a scoping review of their applicability for medication adherence and therapeutic drug monitoring. Drugs.

[9] He N, Su S, Ye Z, Du G, He B, Li D (2020). Evidence-based guideline for therapeutic drug monitoring of vancomycin: 2020 update by the Division of Therapeutic Drug Monitoring, Chinese Pharmacological Society. Clin Infect Dis.

[10] Dervieux T, Greenstein N, Kremer J (2006). Pharmacogenomic and metabolic biomarkers in the folate pathway and their association with methotrexate effects during dosage escalation in rheumatoid arthritis. Arthritis Rheum.

[11] Yang Z, Mo J, Li W, Zhao Z, Mei S (2024). Methotrexate polyglutamates. Expert Rev Clin Pharmacol.

[12] Lucas CJ, Dimmitt SB, Martin JH (2019). Optimising low-dose methotrexate for rheumatoid arthritis: a review. Br J Clin Pharmacol.

[13] Kim H, Kim KS, Kim S, Kang J, Kim HC, Hwang S (2025). Quantitation of methotrexate polyglutamates in red blood cells and application in patients with Crohn’s disease. Transl Clin Pharmacol.

[14] Takahashi C, Kaneko Y, Okano Y, Taguchi H, Oshima H, Izumi K (2017). Association of erythrocyte methotrexate-polyglutamate levels with the efficacy and hepatotoxicity of methotrexate in patients with rheumatoid arthritis: a 76-week prospective study. RMD Open.

[15] Escal J, Poudret M, Hodin S, Neel T, Coman I, Locrelle H (2025). Long-term evaluation of rheumatoid arthritis activity with erythrocyte methotrexate-polyglutamate 3. Fundam Clin Pharmacol.

[16] Kato T, Hamada A, Mori S, Saito H (2012). Genetic polymorphisms in metabolic and cellular transport pathway of methotrexate impact clinical outcome of methotrexate monotherapy in Japanese patients with rheumatoid arthritis. Drug Metab Pharmacokinet.

[17] Shao W, Yuan Y, Li Y (2017). Association between MTHFR C677T polymorphism and methotrexate treatment outcome in rheumatoid arthritis patients: a systematic review and meta-analysis. Genet Test Mol Biomarkers.

[18] He X, Sun M, Liang S, Li M, Li L, Yang Y (2019). Association between ABCB1 C3435T polymorphism and methotrexate treatment outcomes in rheumatoid arthritis patients: a meta-analysis. Pharmacogenomics.

[19] Huang J, Fan H, Qiu Q, Liu K, Lv S, Li J (2020). Are gene polymorphisms related to adverse events of methotrexate in patients with rheumatoid arthritis? A retrospective cohort study based on an updated meta-analysis. Ther Adv Chronic Dis.

[20] Zhang KX, Ip CK, Chung SK, Lei KK, Zhang YQ, Liu L (2020). Drug-resistance in rheumatoid arthritis: the role of p53 gene mutations, ABC family transporters and personal factors. Curr Opin Pharmacol.

[21] Naushad SM, Alrokayan SA, Almajhdi FN, Hussain T (2021). Influence of RFC1 c.80A>G polymorphism on methotrexate-mediated toxicity and therapeutic efficacy in rheumatoid arthritis: a meta-analysis. Ann Pharmacother.

[22] Song Z, Hu Y, Liu S, Jiang D, Yi Z, Benjamin MM (2021). The role of genetic polymorphisms in high-dose methotrexate toxicity and response in hematological malignancies: a systematic review and meta-analysis. Front Pharmacol.

[23] Arida A, Nezos A, Papadaki I, Sfikakis PP, Mavragani CP (2022). Osteoprotegerin and MTHFR gene variations in rheumatoid arthritis: association with disease susceptibility and markers of subclinical atherosclerosis. Sci Rep.

[24] Ceballos FC, Chamizo-Carmona E, Mata-Martín C, Carrasco-Cubero C, Aznar-Sánchez JJ, Veroz-González R (2023). Pharmacogenetic sex-specific effects of methotrexate response in patients with rheumatoid arthritis. Pharmaceutics.

[25] Yamamoto T, Shikano K, Nanki T, Kawai S (2016). Folylpolyglutamate synthase is a major determinant of intracellular methotrexate polyglutamates in patients with rheumatoid arthritis. Sci Rep.

[26] Baghdadi LR, Woodman RJ, Shanahan EM, Wiese MD, Mangoni AA (2018). Genetic polymorphism of the methotrexate transporter ABCG2, blood pressure and markers of arterial function in patients with rheumatoid arthritis: repeated cross-sectional study. Pharmgenomics Pers Med.

[27] Sandhu A, Ahmad S, Kaur J, Bhatnagar A, Dhawan V, Dhir V (2018). Do SNPs in folate pharmacokinetic pathway alter levels of intracellular methotrexate polyglutamates and affect response? A prospective study in Indian patients. Clin Rheumatol.

[28] Ando Y, Shimada H, Matsumoto N, Hirota T, Oribe M, Otsuka E (2013). Role of methotrexate polyglutamation and reduced folate carrier 1 (RFC1) gene polymorphisms in clinical assessment indexes. Drug Metab Pharmacokinet.

[29] Neogi T, Aletaha D, Silman AJ, Naden RL, Felson DT, Aggarwal R (2010). The 2010 American College of Rheumatology/European League Against Rheumatism classification criteria for rheumatoid arthritis: phase 2 methodological report. Arthritis Rheum.

[30] Hanley JA (2016). Simple and multiple linear regression: sample size considerations. J Clin Epidemiol.

[31] Riley RD, Ensor J, Snell KIE, Harrell FE Jr, Martin GP, Reitsma JB (2020). Calculating the sample size required for developing a clinical prediction model. BMJ.

[32] Daraghmeh DN, Moghaddami M, Bobrovskaya L, Proudman SM, Wiese MD (2022). Quantitation of methotrexate polyglutamates in human whole blood, erythrocytes and leukocytes collected via venepuncture and volumetric absorptive micro-sampling: a green LC–MS/MS-based method. Anal Bioanal Chem.

[33] Hawwa AF, Albawab A, Rooney M, Wedderburn LR, Beresford MW, McElnay JC (2014). A novel dried blood spot LC–MS method for the quantification of methotrexate polyglutamates as a potential marker for methotrexate use in children. PLoS One.

[34] Tamai H, Ikeda K, Miyamoto T, Taguchi H, Kuo CF, Shin K (2025). Association of methotrexate polyglutamates concentration with methotrexate efficacy and safety in patients with rheumatoid arthritis treated with predefined dose: results from the MIRACLE trial. Ann Rheum Dis.

[35] van de Meeberg MM, Hebing RCF, Nurmohamed MT, Fidder HH, Heymans MW, Bouma G (2023). A meta-analysis of methotrexate polyglutamates in relation to efficacy and toxicity of methotrexate in inflammatory arthritis, colitis and dermatitis. Br J Clin Pharmacol.

[36] Jara-Palacios MA, Chun W, Traub NL (2021). Potential contributors to low dose methotrexate toxicity in a patient with rheumatoid arthritis and pernicious anemia: case report. BMC Rheumatol.

[37] Shea B, Swinden MV, Ghogomu ET, Ortiz Z, Katchamart W, Rader T (2014). Folic acid and folinic acid for reducing side effects in patients receiving methotrexate for rheumatoid arthritis. J Rheumatol.

[38] Cheng Q, Wu B, Kager L, Panetta JC, Zheng J, Pui CH (2004). A substrate specific functional polymorphism of human gamma-glutamyl hydrolase alters catalytic activity and methotrexate polyglutamate accumulation in acute lymphoblastic leukaemia cells. Pharmacogenetics.

[39] Garcia-Bournissen F, Moghrabi A, Krajinovic M (2007). Therapeutic responses in childhood acute lymphoblastic leukemia (ALL) and haplotypes of gamma glutamyl hydrolase (GGH) gene. Leuk Res.

[40] Zhang HN, He XL, Wang C, Wang Y, Chen YJ, Li JX (2014). Impact of SLCO1B1 521T>C variant on leucovorin rescue and risk of relapse in childhood acute lymphoblastic leukemia treated with high-dose methotrexate. Pediatr Blood Cancer.

[41] Zhang H, He X, Li J, Wang Y, Wang C, Chen Y (2014). SLCO1B1 c.521T>C gene polymorphisms are associated with high-dose methotrexate pharmacokinetics and clinical outcome of pediatric acute lymphoblastic leukemia. Zhonghua Er Ke Za Zhi.

